# Evaluation of antenatal rapid human immunodeficiency virus testing in rural South Africa

**DOI:** 10.4102/sajhivmed.v19i1.771

**Published:** 2018-05-23

**Authors:** Tivani P. Mashamba-Thompson, Pravi Moodley, Benn Sartorius, Paul K. Drain

**Affiliations:** 1 Discipline of Public Health Medicine, School of Nursing and Public Health, University of KwaZulu-Natal, South Africa; 2 Department of Virology, University of KwaZulu-Natal, South Africa; 3 National Health Laboratory Services, Inkosi Albert Luthuli Central Hospital, South Africa; 4 International Clinical Research Center, Department of Global Health, University of Washington, United States; 5 Department of Medicine, University of Washington; Department of Epidemiology, University of Washington, United States; 6 Department of Surgery, Massachusetts General Hospital, Harvard Medical School, United States

## Abstract

**Introduction:**

South African guidelines recommend two rapid tests for diagnosing human immunodeficiency virus (HIV) using the serial HIV testing algorithm, but the accuracy and compliance to this algorithm is unknown in rural clinics. We evaluated the accuracy of HIV rapid testing and the time to receiving test results among pregnant women in rural KwaZulu-Natal (KZN).

**Method:**

We observed the accuracy of rapid HIV testing algorithms for 208 consenting antenatal patients accessing voluntary HIV testing services in nine rural primary healthcare (PHC) clinics in KZN. A PHC-based HIV counsellor obtained finger-prick whole blood from each participant to perform rapid testing using the Advanced Quality™ One Step anti-HIV (1&2) and/or ABON™ HIV 1/2/O Tri-Line HIV test. A research nurse obtained venous blood for an enzyme-linked immunosorbent assay (ELISA) HIV test, which is the gold standard diagnostic test. We recorded the time of receipt of HIV test results for each test.

**Results:**

Among 208 pregnant women with a mean age of 26 years, 72 women from nine rural PHC clinics were identified as HIV-positive by two rapid tests with an HIV-prevalence of 35% (95% Bayesian credibility intervals [BCI]: 28% – 41%). Of the 208 patients, 135 patients from six clinics were tested with the serial HIV testing algorithm. The estimated sensitivity and specificity for the 135 participants were 100% (95% confidence interval [CI]: 93% – 100%) and 99% (CI: 95% – 100%), respectively. The positive predictive value and negative predictive value were estimated at 98% (CI: 94% – 100%) and 95% (CI: 88% – 99%), respectively. All women received their HIV rapid test results within 20 min of testing. Test stock-out resulted in poor test availability at point-of-care, preventing performance of a second HIV test in three out of nine PHC clinics in rural KZN.

**Conclusion:**

Despite the poor compliance with national guidelines for HIV rapid testing services, HIV rapid test results provided to pregnant women in rural PHC clinics in KZN were generally accurate and timely. Test stock-out was shown to be one of the barriers to test availability in rural PHC clinics, resulting in poor compliance with guidelines. We recommend a compulsory confirmation HIV rapid test for all HIV-negative test results obtained from pregnant patients in rural and resource-limited settings.

## Introduction

The World Health Organization’s (WHO) 2016 Consolidated Antiretroviral (ARV) Drugs Guidelines for treating and preventing human immunodeficiency virus (HIV)^1^ and the Joint United Nations Programme on HIV and AIDS (UNAIDS) ‘90-90-90’ strategy^2^ advocate decentralised HIV testing in resource-limited settings. Human immunodeficiency virus (HIV) rapid tests have been a successful intervention to improve healthcare access and outcomes of pregnant women.^3,4,5,6,7,8^ HIV rapid testing can allow timely initiation of antiretroviral therapy (ART) and facilitate linkages to care for HIV-infected women.^3,4,5,6,7,8^ However, to ensure sustainability and accuracy of HIV testing services, HIV rapid tests must meet certain standards.^9^

Human immunodeficiency virus testing is an essential element of antenatal care in South Africa.^10^ The South African Department of Health recently adopted the WHO B+ approach, and recommends ART for all HIV-infected pregnant women.^11,12^ Between 2011 and 2013, there was no change in the antenatal HIV-prevalence.^13^ Substandard diagnostic care and delayed and missed diagnoses have been reported as some of the contributing factors to maternal mortality in rural communities in South Africa.^14^

The South African and WHO guidelines recommend the use of a serial testing algorithm for performing HIV rapid testing in resource-limited settings.^15,16^ The WHO also recommends monitoring the accuracy of HIV rapid tests by comparison to a laboratory-based gold standard HIV test.^16,17^ One of the quality measures is the ability of the test to offer rapid diagnosis to allow the enrolment of HIV-infected pregnant women in prevention of mother-to-child transmission (PMTCT) programmes.^18^ In addition, the WHO recommends that all HIV rapid test results be reported to patients within 30 min of testing.^16^ According to the WHO quality assurance guidelines, HIV-positive status should not be given without two sequential reactive test results in high prevalence (≥ 5%) areas.^19^

Research shows the need for good quality assurance programmes to ensure accuracy of point-of-care (POC) diagnostics in resource-limited settings.^20^ These programmes are essential to ensure that the testing process has been carried out properly and that the test kits and reagents are performing as intended.^15^ The South African rapid HIV testing service guidelines recommend the use of procedural or internal quality control built into the device and independent quality control that is external to the device/kit to improve test accuracy.^15^ The external quality control involves testing of known positive and negative samples which are used to evaluate the accuracy of the test and to check if the person performing the test is performing it correctly.^15^ Our recent study aimed at evaluating the quality management systems, including quality control measures for HIV rapid testing services in rural primary healthcare (PHC) clinics in KwaZulu-Natal, revealed that there is a need for improving quality control measures for rapid HIV testing, particularly staff competency.^21^ However, this evaluation study did not include assessment of HIV test accuracy. It has been recommended that the accuracy of rapid HIV tests should be evaluated by the actual test user and in the appropriate clinical settings.^22^ As there has been limited reporting on the accuracy of HIV rapid tests in rural and resource-limited settings in South Africa, we sought to evaluate the accuracy and performance of HIV rapid test results and the time to report the HIV rapid test result to pregnant women in rural South Africa.

## Material and methods

### Study design

This manuscript was produced as part of a large research study entitled ‘Evaluating the accessibility and utility of HIV-related point-of-care diagnostics for maternal health in rural South Africa’.^23^ The large study included a survey of 100 clinics aimed at determining the accessibility, availability and usage of POC diagnostic tests in PHC clinics in rural KZN. Multistage sampling was conducted in this study. The initial sampling stage involved proportional stratified sample of 100 clinics from all 11 districts in KZN to ensure generalisability of the survey results. HIV rapid tests were shown to be the most universally available and used test in the participating clinics. In order to determine the accuracy of the results produced from the HIV POC diagnostics services in PHC clinics in rural KZN PHC clinics, we conducted a cross-sectional study of pregnant women among nine antenatal clinics in rural PHC clinics (Table 1). All participating clinics were located within 60 km of the testing laboratory and were part of large POC diagnostic survey study.^23^ The catchment areas for participating clinics consisted of rural and resource-limited communities.

**TABLE 1:** Characteristics of the participating patients.

District	Facility	Monthly patient census (Median [IQR])	Sample size	Patient age (Median [IQR])	Monthly HIV-positive results (Median [IQR])
eThekwini MM	Adams Mission Clinic	3321.5 (IQR = 3665)	11	25 (IQR = 5)	29.47 (IQR = 17.49)
eThekwini MM	Danganya Clinic	9382 (IQR = 4615)	26	24.5 (IQR = 10)	17.52 (IQR = 10.81)
eThekwini MM	Fredville Clinic	9403 (IQR = 676)	27	24 (IQR = 13)	4.17 (IQR = 14.27)
eThekwini MM	Umbumbulu Clinic	10 657 (IQR = 4161.5)	56	24 (IQR = 9)	17.72 (IQR = 5.48)
eThekwini MM	Magabheni Clinic	6521 (IQR = 3020.5)	20	25.5 (IQR = 5.5)	11.81(IQR = 7.88)
eThekwini MM	Msunduze Bridge Clinic	11 856 (IQR = 926)	35	28 (IQR = 11)	16.67 (IQR = 8.97)
eThekwini MM	Ntshongweni Clinic	3935.5 (IQR = 1711)	11	22 (IQR = 4)	21.42 (IQR = 19.87)
uMgungundlovu DM	Gcumisa Clinic	5713 (IQR = 775)	17	24 ( IQR = 11)	24.04 (IQR = 7.94)
uMgungundlovu DM	Mbuthisweni Clinic	1561 (IQR = 206.5)	5	25 (IQR = 17)	13.23 (IQR = 19.81)

Antenatal nurses providing antenatal services assisted with identification of potential study participants and referred them to the research team. After informing the women about the purpose of the study, consenting women were screened for participation. We included pregnant women 18 years of age and older. Consenting patients received voluntary HIV counselling and testing conducted by trained HIV counsellors who were part of PHC clinics’ staff members at no additional cost and as part of standard antenatal services. The HIV rapid testing was carried out as part of the routine antenatal clinic service. PHC clinic-based HIV lay counsellors met privately with each patient, obtained a sexual history and discussed the risks as well as benefits of HIV testing and obtained informed consent before conducting the HIV rapid tests. All patients were offered post-test counselling. Those who tested HIV-positive on HIV rapid tests were referred for ART initiation and PMTCT services, which were provided as part of standard antenatal services.

### Human immunodeficiency virus rapid testing methods

The two HIV rapid tests used by the clinics were Advanced Quality™ One Step Anti-HIV 1&2 (InTec Products, Inc. Xiamen, China) and ABON™ HIV 1/2/O Tri-Line lateral immunoassays rapid test (ABON Biopharm Co. Ltd. Hangzhou, China). The HIV rapid tests were performed according to the manufacturer’s package inserts and the results were interpreted by trained HIV counsellors. The South African rapid HIV testing service guidelines recommend that rural PHC clinics perform regular quality control to ensure accurate HIV rapid testing.^15^ Internal quality control built into the device and external quality control for HIV rapid tests are performed in the clinics.

**FIGURE 1:**
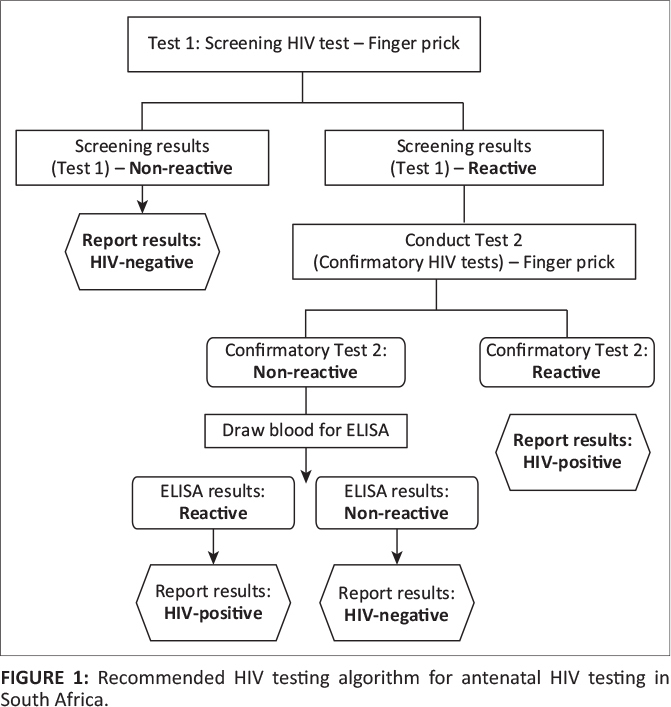
Recommended HIV testing algorithm for antenatal HIV testing in South Africa.

To assess the accuracy of HIV rapid testing services in rural PHC clinics, clinic-based nurses and HIV lay counsellors performed serial HIV rapid testing as recommended by the national HIV rapid testing guidelines^24^ (Figure 1). Using the serial HIV rapid testing algorithm, each patient was tested using the Advanced Quality™ HIV rapid test, and those who tested positive were then retested using the ABON™ HIV rapid test. If the first test was positive, then the second HIV rapid test was performed. If the first HIV test was negative, the second test was not performed and participants were counselled to return in three months for repeat HIV testing. Where patients tested positive on first test and negative on second test, a third test was provided using ABON rapid test. The HIV testing procedure stipulated that a 20-min timer should be set after adding drawn whole blood specimen (about 50 µL) from the patient. The blood is then immersed onto the specimen well of the test device, followed by two drops of buffer (approximately 80 µL).

### Laboratory methods

For all participants, a professional research nurse obtained at least 5 mL of venous whole blood in a serum separator tube for HIV ELISA testing. Laboratory personnel were blinded to clinic HIV rapid test results. The tube was kept on ice before being transported within 3 h of being collected to the Department of Virology in the National Health Laboratory Services (NHLS) at Inkosi Albert Luthuli Central Hospital (IALCH) in Durban.

A laboratory technician centrifuged the specimen at 10 000 rpm for 5 min and stored the serum sample at 2 °C – 8 °C before testing. Upon HIV ELISA testing, sera were thawed to room temperature, and HIV ELISA testing was performed using the Combi PT HIV-1/2 Antigen and total antibodies using a cobas^®^ e601 machine (Roche Diagnostics, Manneheim, Germany).^16^ All positive ELISA specimens underwent confirmatory HIV ELISA testing using Vironostika HIV Ag/Ab Microelisa System (bioMerieux, Lyon, France). The HIV ELISA tests were performed and interpreted by trained laboratory personnel. All quality assurance procedures were included, by using manufacturer’s internal quality control and a laboratory that is SANAS ISO 15189 accredited. The laboratory participates in an external proficiency-testing scheme from the NHLS (Sandringham, South Africa). Any discordant results between HIV rapid test and ELISA were reported to the antenatal clinic within a week of testing and the patients were recalled for a confirmation test.

### Statistical methods

We calculated the sample size for the evaluation at individual participant level using Buderer’s formula.^25^ The results from each single rapid HIV test were analysed separately. Our estimated sample size of 205 participants was based on assuming an absolute precision of ± 10% and the prevalence of disease in the study population is 27%.^26^ A proportionate representative patient sample size was calculated based on average PHC clinic average weekly patient census, obtained from the 2014 South African District Health Information Software (DHIS).

The accuracy of the 208 HIV RT results from the selected rural PHC antenatal clinics was evaluated using serial (where a second test is required only when the initial test is positive) or single testing algorithms. We used R version 3.2.3 (2015), CRAN bdpv-package,^27^ for calculating sensitivity, specificity, positive likelihood ratio (+LR) and negative likelihood ratio (-LR), as well as positive predictive value (PPV) and negative predictive value (NPV) analyses. Confidence intervals (CI) of 95% were estimated for sensitivity, specificity, +LR and -LR, as well as PPV and NPV. Disease prevalence was analysed using Bayesian statistics, and 95% Bayesian credibility intervals (BCI) were estimated. Our model was fitted using Markov chain Monte Carlo simulation. Posterior distributions of the parameters were obtained using WinBUGS software.^28^ Model convergence was assessed by visual inspection of the parameter series plots based on Gelman-Rubin statistics (Appendix 1). Once convergence was achieved, the chains were then sampled until a sample size of 10 000 iterations were attained to estimate the final parameter point estimates and 95% BCI for HIV-prevalence.

## Ethical consideration

Ethical approvals for the study were received from the KZN Department of Health’s Ethics Committee (HRKM 40/15) and the University of KZN Biomedical Research Ethics Committee (BE484/14). Written informed consent was obtained from all study participants.

## Results

We enrolled 208 pregnant women from nine rural antenatal clinics with a total monthly census ranging from 18 794 to 140 152 patients. The age of the participants ranged from 18 to 51 years. The estimated HIV-prevalence for the sampled population was 34.5% (BCI: 28.3% – 41.1%). A summary of the study population is presented in Table 1.

### Accuracy of serial human immunodeficiency virus rapid testing

**FIGURE 2:**
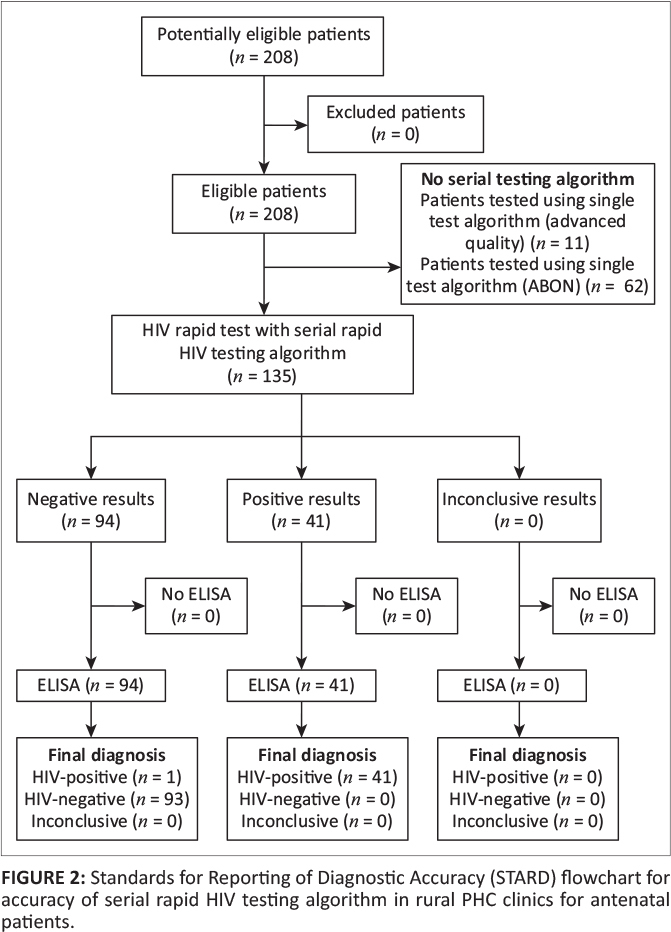
Standards for Reporting of Diagnostic Accuracy (STARD) flowchart for accuracy of serial rapid HIV testing algorithm in rural PHC clinics for antenatal patients.

A total of 135 (65%) antenatal specimens from six out of nine PHC clinics were tested by the HIV rapid testing serial algorithm, and the results were compared to the results of the serum ELISA reference testing. The diagnostics tests accuracy results are illustrated by the Standards for Reporting of Diagnostic Accuracy (STARD) flowchart (Figure 2).

The Advanced Quality HIV test was used for an initial screening of 135 tests and initially detected 41 HIV antibody-reactive specimens and 83 antibody-negative reactions, as well as one false-negative. The one false-negative result was tested using the Advanced Quality HIV rapid screening test, following the serial HIV rapid testing algorithm, which stipulated no obligation for a confirmation test for HIV-negative results from the screening test. Secondary testing of the initial reactive samples was performed using ABON (*n* = 49). A total of 41 HIV antibody-reactive samples were found using ABON as a second test. A total of 10 samples were tested by ABON test as a confirmation of the previous HIV antibody non-reactive test by Advanced HIV RT. The estimated sensitivity, specificity, PPV and NPV are shown in Table 2.

**TABLE 2:** Diagnostic accuracy of human immunodeficiency virus rapid testing among pregnant women attending antenatal clinics in KwaZulu-Natal.

Sample type	Pregnant women tested	Sensitivity (95% CI)		Specificity (95% CI)		PPV (95% CI)	NPV (95% CI)
Entire cohort	208	(71/71)	100% (96% –100%)	(136/137)	99% (96% – 99%)	99% (96% –100%)	97% (89% – 99%)
Samples tested using serial HIV rapid test algorithm (Advanced Quality and ABON)	135	(41/41)	100% (93% –100%)	(93/94)	99% (95% – 100%)	98% (94% –100%)	95% (88% – 98%)
Samples tested using single test algorithm (Advanced Quality)	11	(4/4)	100% (0.47% – 100%)	(7/7)	100% (65% –100%)	89% (69% – 96%)	75% (44% – 90%)
Samples tested using single test algorithm (ABON)	62	(26/26)	100% (89% –100%)	(36/36)	100% (92% –100%)	97% (91% – 99%)	93% (78% – 98%)

CI, confidence interval; NPV, negative predictive value; PPV, positive predictive value.

### Compliance to the human immunodeficiency virus testing guidelines

The routine serial testing algorithm with a screen and confirmatory rapid test has been recommended for use in the health clinics.^15,16^ However, the use of this algorithm is dependent on test availability; three of the nine participating PHC clinics (Adams, Fredville and Mzunduze Bridge) were experiencing test shortages owing to stock-outs and therefore they offered only one HIV rapid test (ABON or Advanced Quality) for the diagnosis of HIV.

The Advanced Quality HIV rapid test was the only HIV rapid test offered to antenatal patients in Adams clinic (*n* = 11) constituting 5.3% of the total sample size. Because of a shortage of the ABON HIV rapid test, four HIV antibody-reactive specimens and nine antibody-negative reactions were identified. The ABON rapid test was used as the only HIV rapid test for 62 (29.8%) of the participants from Fredville and Msunduze Bridge clinic, owing to a shortage of the Advanced Quality HIV rapid test, and 27 HIV antibody-reactive specimens and 35 antibody-negative reactions were identified.

### Time of results receipt

Following the pre-test counselling, which includes training the patient on how to accurately interpret the HIV rapid test results, all HIV rapid test results were read to the participants within 20 min.

## Discussion

In this cross-sectional study of rural antenatal clinics in KwaZulu-Natal, the HIV rapid tests using the serial HIV rapid testing algorithm were accurate when performed by nurses and HIV counsellors at the clinical POC.^16^ However, owing to shortages of rapid HIV tests, only 65% of women were tested using the recommended HIV rapid testing algorithm.^24^ In our study, only one false-negative was reported out of 135 tested using the Advanced Quality rapid test under the recommended serial testing strategy. According to the serial HIV rapid testing algorithm, a second test is not mandatory for samples that test negative on the first test. As a result, the false-negative test was not confirmed with a second test at POC. The overall diagnostic accuracy and time of receipt of HIV rapid test results met the WHO recommendations for an ideal HIV rapid testing service in resource-limited settings.^16^ In our limited sample size, using a single HIV screening test was shown to be more accurate than the serial screening testing algorithm. All HIV rapid test results were reported within 20 min.

The findings of this study support the results of a recent (2017) study which was aimed at evaluating the quality management systems of HIV rapid testing services in rural PHC clinics in KwaZulu-Natal.^21^ The evaluation study showed that there is poor quality supply chain management and poor adherence to standards among staff in rural PHC clinics.^21^ These findings are also supported by Mbachu et al.,^29^ Pavie et al.^30^ and Moodley et al.,^31^ who showed the accuracy of HIV rapid testing services in resource-limited settings. To ensure sustainable accuracy of the HIV rapid test services provided, it is important that evidence-based guidelines are followed, particularly in high HIV-prevalence regions. Optimal HIV testing and counselling strategies are crucial for improvement of maternal outcomes and PMTCT of HIV in resource-limited settings. The WHO quality-ASSURED (**A**ffordable, **S**ensitive, **S**pecific, **U**ser friendly, **R**apid to enable treatment at first visit and robust, **E**quipment free and **D**elivered to those who need it) criteria recommend that the HIV rapid test has a sensitivity of 99% and specificity of 99%.^16^ The WHO also recommends the following predictive values for high (> 30%) HIV-prevalence settings: 98% PPV with one reactive test; 100% PPV with two reactive tests and; 99.6% NPV with one non-reactive test.^16^

Our study included nine rural antenatal clinics across a large province in South Africa. We have demonstrated that the use of one HIV rapid test offers quick and reliable HIV results for HIV diagnosis to allow linkage to ART and PMTCT services for HIV-infected pregnant women at their first visit to antenatal clinics. Sample mismatch and sample loss have been reported as some of the pre-analytical errors that can occur during sample transportation from the POC to the hospital-based laboratory.^32^ The one false-positive result obtained from the laboratory ELISA test demonstrates an increased likelihood of a sample mismatch or sample loss during transportation from testing site to laboratory. These findings also support the advantage of using HIV rapid testing for pregnant women in addition to other previously reported advantages of POC diagnostics over standard laboratory testing.^33,34^ Poor compliance to standard protocols by healthcare providers has been listed among the most common causes of maternal deaths in South Africa.^35^ Strategies aimed at improving supply chain maintenance and healthcare workers’ compliance to HIV rapid test standards are required to ensure continual accuracy of the HIV rapid testing provided to pregnant women in rural and resource-limited settings. In addition, continual training courses for HIV counsellors in rural and resource-limited settings are recommended. Bearing in mind the importance of PMTCT for HIV, the prevalence of HIV and the current level of healthcare accessibility in rural KZN, we recommend a revision of the National HIV Counselling and Testing Policy Guidelines^24^ to include a compulsory confirmation HIV rapid test for HIV-negative results for pregnant women in rural and resource-limited settings. This testing should be conducted during the same clinic visit at POC, prior to the repeat HIV testing on every scheduled visit, during labour and through breastfeeding every three months. Results of this study show acceptability of the single test HIV rapid testing algorithm. Therefore, in cases where availability of HIV rapid testing is poor and only one test can be made available, we recommend the use of the ABON test or Advanced Quality test for screening and confirmation of the test results.

## Limitations

Our study had several strengths and limitations. One of the unavoidable limitations is the availability of the HIV POC tests which prevented 35% of our study population from following the recommended serial rapid HIV testing algorithm, leading to a reduction in sample size used to report accuracy, from 208 to 135 patients. In this study, data on CD4+ count or duration of pregnancy were not collected; this information would have provided more nuance information about the patients to enable early detection of ARV resistance and HIV co-infections. Furthermore, this study excluded remote rural antenatal clinics outside the 60 km radius of the testing laboratory. The more remote clinics may have greater difficulties in stocking rapid HIV tests and more difficulty accessing laboratory testing. Therefore, our findings may not be generalisable to more remote (> 60 km from the testing laboratory) rural PHC clinics in KZN. Other important quality control criteria such as quality systems^36^ required to implement quality management, including activities which contribute directly or indirectly to the quality of tests of HIV rapid testing, were not evaluated. A parallel study was conducted to access quality systems management for HIV rapid testing services in rural KZN.^21^

## Conclusion

Human immunodeficiency virus rapid testing services can be accurately performed at the clinical POC for pregnant women in resource-limited settings, and we suggest that one HIV rapid test should be sufficient. If viable, then moving to a single rapid HIV test algorithm has the potential to save limited resources. This study also demonstrated HIV rapid tests stock-outs in KZN rural PHC clinics. Based on these findings, further efforts to optimise the availability of HIV rapid testing services in settings with poor access to laboratory infrastructure are needed.

## Appendix 1

### The winbugs code

model{

y[1:4]~dmulti(p[1:4],n)

p[1]<-pi*S1*S2+(1-pi)*(1-C1)*(1-C2)

p[2]<-pi*S1*(1-S2)+(1-pi)*(1-C1)*C2

p[3]<-pi*(1-S1)*S2+(1-pi)*C1*(1-C2)

p[4]<-pi*(1-S1)*(1-S2)+(1-pi)*C1*C2

#Prior distribution s

pi~dbeta(1,1)

S1~dbeta(1,1)

C1~dbeta(1,1)

S2~dbeta(1,1)

C2~dbeta(1,1)

PPV2<-S2*pi/(S2*pi+(1-C2)*(1-pi))

NPV2<-C2*(1-pi)/((1-S2)*pi+C2*(1-pi))

}

list(n=208,y=c(71,0,1,136))

list(pi=0.4,S1=0.9,C1=0.9,S2=0.9,C2=0.9)

## References

[1] World Health Organization. Consolidated guidelines on the use of antiretroviral drugs for treating and preventing HIV infection: Recommendations for a public health approach [homepage on the Internet]. 2016 [cited 2016 Aug 12]. Available from: http://apps.who.int/iris/bitstream/10665/208825/1/9789241549684_eng.pdf?ua=1 27466667

[2] UNAIDS. 90-90-90: An ambitious treatment target to help end the AIDS epidemic [homepage on the Internet]. 2014 [cited 2016 Nov 30]. Available from: http://www.unaids.org/en/resources/documents/2017/90-90-90

[3] Heller T, Kunthea S, Bunthoeun E, et al. Point-of-care HIV testing at antenatal care and maternity sites: Experience in Battambang Province, Cambodia. Int J STD AIDS. 2011;22(12):742–747. 10.1258/ijsa.2011.011262 22174058

[4] Kissin DM, Akatova N, Rakhmanova AG, et al. Rapid HIV testing and prevention of perinatal HIV transmission in high-risk maternity hospitals in St. Petersburg, Russia. Am J Obstet Gynecol. 2008;198(2):183 e181–187. 10.1016/j.ajog.2007.09.005 18226620

[5] Dennis RL, Negron TJ, Lindsay M, Nesheim SR, Lee FK, Jamieson DJ. Rapid human immunodeficiency virus testing in labor and delivery: A comparison of implementation models between 2 hospitals. J Perinat Neonatal Nurs. 2007;21(4):298–306. 10.1097/01.JPN.0000299787.24291.8d 18004167

[6] Kizito D, Woodburn PW, Kesande B, et al. Uptake of HIV and syphilis testing of pregnant women and their male partners in a programme for prevention of mother-to-child HIV transmission in Uganda. Trop Med Int Health. 2008;13(5):680–682. 10.1111/j.1365-3156.2008.02052.x 18331533 PMC2592475

[7] Nogueira SA, Lambert JS, Albuquerque AL, et al. Assessment of a rapid HIV test strategy during labor: A pilot study from Rio de Janeiro, Brazil. J Hum Virol. 2000;4(5):278–282. 11907385

[8] Smith A, Sabido M, Camey E, Batres A, Casabona J. Lessons learned from integrating simultaneous triple point-of-care screening for syphilis, hepatitis B, and HIV in prenatal services through rural outreach teams in Guatemala. Int J Gynaecol Obstet. 2015;130(Suppl 1):S70–S72. 10.1016/j.ijgo.2015.04.009 25968489

[9] Shott JP, Galiwango RM, Reynolds SJ. A quality management approach to implementing point-of-care technologies for HIV diagnosis and monitoring in sub-Saharan Africa. J Trop Med. 2012;2012:1–8. 10.1155/2012/651927 PMC3263631 22287974

[10] Barron P, Pillay Y, Doherty T, et al. Eliminating mother-to-child HIV transmission in South Africa. Bull World Health Organ. 2013;91(1):70–74. 10.2471/BLT.12.106807 23397353 PMC3537246

[11] TM Publications. Point of care diagnostics testing world markets. [homepage on the Internet]. 2013 [cited 2016 Aug 14]. Available from: https://www.prnewswire.com/news-releases/point-of-care-diagnostic-testing-world-markets-300358818.html

[12] Garvey M, Garvey AJ. Philosophy and opinions of Marcus Garvey or Africa for the Africans. Philosophy and opinions of Marcus Garvey, or, Africa for the Africans. London: Frank Cass & Co., Ltd.; 1967.

[13] Khan M, Pillay T, Moodley JM, Connolly CA, Group DPTH-S. Maternal mortality associated with tuberculosis–HIV-1 co-infection in Durban, South Africa. AIDS. 2001;15(14):1857–1863. 10.1097/00002030-200109280-00016 11579249

[14] Amnesty International. Struggle for maternal health barriers to antenatal care in South Africa [homepage on the Internet]. 2014 [cited 2016 Aug 12]. Available from: https://www.health-e.org.za/wp-content/uploads/2014/10/Struggle-for-Maternal-Health-.pdf

[15] Department of Health South Africa. Guidelines for assuring the accuracy and reliability of HIV rapid testing [homepage on the Internet]. 2009 [cited 2016 Aug 12]. Available from: http://policyresearch.limpopo.gov.za/bitstream/handle/123456789/897/Guidelines%20for%20assuring%20the%20accuracy%20and%20reliability%20of%20HIV%20rapid%20testing%2C.pdf?sequence=1

[16] World Health Organization. Rapid HIV tests: Guidelines for use in HIV testing and counselling services in resource-constrained settings [homepage on the Internet]. 2004 [cited 2016 Nov 14]. Available from: http://apps.who.int/iris/bitstream/10665/42978/1/9241591811.pdf

[17] Crippen P, Demby A, Miller D, Vercauteren G, World Health Organization. Rapid HIV tests: Guidelines for use in HIV testing and counselling services in resource-constrained settings. Switzerland: World Health Organization, Department of HIV/AIDS; 2004. Available from: http://applications.emro.who.int/aiecf/web28.pdf

[18] Peeling RW, Mabey D, Herring A, Hook EW. Why do we need quality-assured diagnostic tests for sexually transmitted infections? Nat Rev Microbiol. 2006;4(12):909–921. 10.1038/nrmicro1555 17109030

[19] World Health Organization. Annex 9: Technical guidance update on quality assurance for HIV rapid diagnostic tests. 2015 [cited 12 Nov 12]. Available from: http://apps.who.int/iris/bitstream/10665/181244/1/WHO_HIV_2015.28_eng.pdf

[20] Yager P, Domingo GJ, Gerdes J. Point-of-care diagnostics for global health. Annu Rev Biomed Eng. 2008;10:107–144. 10.1146/annurev.bioeng.10.061807.160524 18358075

[21] Jaya Z, Drain PK, Mashamba-Thompson TP. Evaluating quality management systems for HIV rapid testing services in primary healthcare clinics in rural KwaZulu-Natal, South Africa. PLoS One. 2017;12(8):e0183044. 10.1371/journal.pone.0183044 28829801 PMC5567898

[22] Banoo S, Bell D, Bossuyt P, et al. Evaluation of diagnostic tests for infectious diseases: General principles. Nat Rev Micro. 2008;6:S16–S28. 10.1038/nrmicro1523 22745954

[23] Mashamba-Thompson T, Drain P, Sartorius B. Evaluating the accessibility and utility of HIV-related point-of-care diagnostics for maternal health in rural South Africa: A study protocol. BMJ Open. 2016;6(6):e011155. 10.1136/bmjopen-2016-011155 PMC4932288 27354074

[24] Department of Health South Africa. National HIV counselling and testing policy guidelines [homepage on the Internet]. 2015 [cited 2016 Nov 30]. Available from: https://www.health-e.org.za/wp-content/uploads/2015/07/HCT-Guidelines-2015.pdf

[25] Buderer NMF. Statistical methodology: I. incorporating the prevalence of disease into the sample size calculation for sensitivity and specificity. Acad Emerg Med 1996;3(9):895–900. 10.1111/j.1553-2712.1996.tb03538.x 8870764

[26] Burd EM. Validation of laboratory-developed molecular assays for infectious diseases. Clin Microbiol Rev. 2010;23(3):550–76. 10.1128/CMR.00074-09 20610823 PMC2901657

[27] Adachi K, Klausner JD, Xu J, et al. Chlamydia trachomatis and Neisseria gonorrhoeae in HIV-infected pregnant women and adverse infant outcomes. Pediatr Infect Dis J. 2016;35(8):894–900. 10.1097/INF.0000000000001199 27164464 PMC4945428

[28] Joseph L, Gyorkos TW, Coupal L. Bayesian estimation of disease prevalence and the parameters of diagnostic tests in the absence of a gold standard. Am J Epidemiol. 1995;141(3):263–272. 10.1093/oxfordjournals.aje.a117428 7840100

[29] Mbachu II, Udigwe G, Joseph I, et al. The evaluation of accuracy of serial rapid HIV test algorithm in the diagnosis of HIV antibodies among pregnant women in south east Nigeria. BMC Res Notes. 2015;8(1):557. 10.1186/s13104-015-1454-8 26459010 PMC4603774

[30] Pavie J, Rachline A, Loze B, et al. Sensitivity of five rapid HIV tests on oral fluid or finger-stick whole blood: A real-time comparison in a healthcare setting. PLoS One. 2010;5(7):e11581. 10.1371/journal.pone.0011581 20657834 PMC2906506

[31] Moodley D, Moodley P, Ndabandaba T, Esterhuizen T. Reliability of HIV rapid tests is user dependent. S Afr Med J. 2008;98(9):707–709. 19113051

[32] Carey M, Markham C, Gaffney P, Boran G, Maher V. Validation of a point of care lipid analyser using a hospital based reference laboratory. Irish J Med Sci. 2006;175(4):30–35. 10.1007/BF03167964 17312826

[33] Pai NP, Vadnais C, Denkinger C, Engel N, Pai M. Point-of-care testing for infectious diseases: Diversity, complexity, and barriers in low- and middle-income countries PLoS Med. 2012;9:e1001306. 10.1371/journal.pmed.1001306 22973183 PMC3433407

[34] Price CP. Point of care testing. Br Med J. 2001;322(7297):1285. 10.1136/bmj.322.7297.1285 11375233 PMC1120384

[35] Ott JJ, Stevens GA, Wiersma ST. The risk of perinatal hepatitis B virus transmission: Hepatitis B e antigen (HBeAg) prevalence estimates for all world regions. BMC Infect Dis. 2012;12(1):1. 10.1186/1471-2334-12-131 22682147 PMC3478174

[36] Dybkaer R, Jordal R, Jørgensen P, et al. A quality manual for the clinical laboratory including the elements of a quality system: Proposed guidelines. Scand J Clin Lab Invest. 1993;53(Suppl 212):60–77. 10.1080/00365519309085460 8465158

